# Activated Carbon and Biochar Derived from *Sargassum* sp. Applied in Polyurethane-Based Materials Development

**DOI:** 10.3390/polym16202914

**Published:** 2024-10-16

**Authors:** Julie Mallouhi, Miklós Varga, Emőke Sikora, Kitty Gráczer, Olivér Bánhidi, Sarra Gaspard, Francesca Goudou, Béla Viskolcz, Emma Szőri-Dorogházi, Béla Fiser

**Affiliations:** 1 Institute of Chemistry, University of Miskolc, 3515 Miskolc-Egyetemváros, Hungary; julie.mallouhi@gmail.com (J.M.); miklos.varga@uni-miskolc.hu (M.V.); emoke.sikora@uni-miskolc.hu (E.S.); kitti.graczer@uni-miskolc.hu (K.G.); oliver.banhidi@uni-miskolc.hu (O.B.); bela.viskolcz@uni-miskolc.hu (B.V.); 2 Higher Education and Industrial Cooperation Centre, University of Miskolc, 3515 Miskolc-Egyetemváros, Hungary; 3 Laboratory COVACHIM-M2E, EA 3592 Université des Antilles, BP 250, 97157 Pointe à Pitre, Cedex, France; sarra.gaspard@univ-antilles.fr (S.G.); francesca.goudou@univ-antilles.fr (F.G.); 4 Department of Biology and Chemistry, Ferenc Rakoczi II Transcarpathian Hungarian College of Higher Education, 90200 Beregszász, Ukraine; 5 Department of Physical Chemistry, Faculty of Chemistry, University of Lodz, 90-236 Lodz, Poland

**Keywords:** polyurethane, polymer composite, acoustic properties, toxicity test

## Abstract

Activated carbon (AC) and biochar (BC) are porous materials with large surface areas and widely used in environmental and industrial applications. In this study, different types of AC and BC samples were produced from *Sargassum* sp. by a chemical activation and pyrolysis process and compared to commercial activated carbon samples. All samples were characterized using various techniques to understand their structure and functionalities. The metal content of the samples was characterized by using an inductively coupled optical emission spectrometer (ICP-OES). A toxicity test was applied to investigate the effect of AC/BC on organisms, where *Sinapis alba* seed and *Escherichia coli* bacteria-based toxicity tests were used. The results revealed that the samples did not negatively affect these two organisms. Thus, it is safe to use them in various applications. Therefore, the samples were tested as fillers in polyurethane composites and, thus, polyurethane-AC/BC samples were prepared. The amounts of AC/BC mixed into the polyurethane formulation were 1%, 2%, and 3%. Mechanical and acoustic properties of these composites were analyzed, showing that by adding the AC/BC to the system an increase in the compression strength for all the samples was achieved. A similar effect of the AC/BC was noticed in the acoustic measurements, where adding AC/BC enhanced the sound adsorption coefficient (α) for all composite materials.

## 1. Introduction

Activated carbon (AC) is a porous carbonaceous material with pores of varying sizes [1]. It is widely used in many different industrial applications [2,3] due to its large specific surface area, capacity to reactivate, desirable pore structure, strong chemical resistance, and the presence of various oxygen-containing functional groups on the surface [4]. There is an increasing demand for AC and, thus, its production has to be cheap and efficient. Activated carbon can be made from any carbonaceous substance, but the starting materials will define the cost and other properties of the final product. To achieve cost-effective production of AC, agricultural by-products which are easily available and abundant can be used as raw materials. Accordingly, wood, waste nut shells, cherry stones, tea waste, sugarcane bagasse, or fruit pits can be used as AC base material, and they are more cost-effective than the production of granular AC from non-renewable coal [2,5].

In Guadeloupe (Caribbean), it is a big problem that a brown macroalga called *Sargassum* sp. grows in huge quantities on the shores [6,7,8,9]. This is quite problematic because ammonia and hydrogen sulfide gases can be released during the macroalga’s decomposition, and thus, the overgrowth of *Sargassum* sp. is causing several concerns, including health problems [7]. Additionally, because it has an indirect impact on tourism, it has negative economic effects as well. Thus, by using *Sargassum* sp. as raw materials for AC production, the negative effects of the overgrowth of the macroalga can be turned into an advantage and valuable products can be achieved [6,7].

The most important step in the production of activated carbon is activation. There are two methods commonly used to produce AC: physical activation and chemical activation, where the choice of method depends on the starting material and the desired carbon form (powdered, granular, or low-density) [2]. During the chemical activation, chemicals that usually act as drying agents and encourage pyrolytic decomposition such as phosphoric acid (H_3_PO_4_) are used which can reduce the formation of tar during the pyrolysis process and increase the pores in activated carbon [2].

Besides activated carbon, another type of possible carbonaceous material is biochar (BC), which is a porous composition primarily comprising carbon [10]. Biochar is produced by pyrolysis of biomass such as wood, grass, peanut straw, crop residues, or *Sargassum* sp. [11,12,13,14]. Pyrolysis is a thermal decomposition process that involves the presence of little or no oxygen and occurs at temperatures ranging from 300 to 800 °C [15].

These porous materials with a large specific surface area have attracted much interest [3]. Activated carbon and biochar could be used to produce polymer composites and by the introduction of AC or BC into the polymeric materials the mechanical and acoustic properties can be enhanced [16,17,18].

One of the most common types of polymer is polyurethane foam (PUF) with many applications in diverse industries [19] such as furniture, thermal insulation materials, automotives, sound-insulating materials, lightweight structural materials, and vibration-damping materials due to its low cost, low density, easy manufacturing processes, and good mechanical properties [20,21,22]. PUFs are normally produced by polymerization between polyols and isocyanates along with additives [23]. To improve the PUF’s characteristics, it is common to produce composite material by adding filler to the polymer matrix [19,23]. In this respect, there are a variety of fillers, including various carbonaceous materials, that are capable of fulfilling a particular purpose [17,20].

There are studies on using biochar as filler, where increasing the amount of BC in polyurethane foam reduced the compressive strengths at 10% deformation [15]. Various forest waste fillers (wood, bark, cones, and needles from pine trees, kraft lignin, and recycled paper sludge from industry wastes) were added to polyurethane foam in different amounts (1, 5, and 10 wt%) with NCO/OH ratios (0.6, 0.9, and 1.2), and it was observed that the compositions reinforced with 1 wt% and 5 wt% wood were the most effective, demonstrating superior mechanical performance. This enhancement is likely due to the wood’s increased PU system compatibility, which promotes the isocyanate and filler’s urethane bond formation [24].

Furthermore, flexible polyurethane foams (FPUFs) can be utilized for sound insulation. Because of the presence of pores and cavities in the structure, they are effective at absorbing sound energy, making them suitable for noise control [23]. Composite polyurethane (PU) foams enhanced with highly nanoporous activated carbon (AC) have been investigated as an eco-friendly sound-absorbing material with exceptional broadband sound absorption properties, where it was observed that the enhanced composite material achieved a 95.8% absorption of incident acoustic waves within the 2000–5000 Hz frequency range. This performance significantly surpasses that of pristine PU foam, which only absorbs 70.6% in the same range [25].

Despite activated carbon and biochar being highly efficient materials utilized in various applications, there are concerns regarding their possible toxicity [26]. Toxic materials can be released from them due to the fact that heavy metals or other toxic compounds can be present in the original matrix utilized to prepare activated carbon or biochar [10], thus, since these materials can be used in various applications such as adsorbing pollutants in water and soil amendment, it is important to know how the materials can affect organisms in the environment. To evaluate the potential adverse effects of these materials on organisms, toxicity tests are carried out. To effectively assess the environmental risks associated with activated carbon/biochar usage, it is crucial to predict potential side effects and utilize exposure bioindicators in the risk assessment process [27]. To assess toxicity, a variety of bioassays are performed using sensitive species including fish, rats, and algae. Despite their efficacy, these techniques have several drawbacks, including high costs and the need for specific equipment and knowledge, and can take a considerable amount of time to produce results [28]. The germination test is a short-term assessment technique used in ecotoxicological evaluations to determine acute toxicity by measuring the rate of germination and root length of seeds exposed to different concentrations of a test substance [10,29]. Previous studies have noted that activated carbon and biochar can have varying effects on microorganisms and plants, depending on the specific organism or strain involved [10].

A lot of research has focused on the production and utilization of activated carbon and biochar derived from agricultural wastes and renewable resources to develop eco-friendly and cost-effective solutions for various applications such as water purification [2,5] or an adsorbent for pollutant sequestration and soil amendment. Their incorporation into soil has been shown to improve various physical, chemical, and biological properties, enhancing soil fertility and ultimately promoting plant growth and productivity. Additionally, they have a good effect on soil enzymatic activity, making the environment more suitable for beneficial microbes and improving plant health [30].

For instance, activated carbon derived from coconut shells and biochar derived from biomass (wood residues) were tested on spinach (*Spinacia oleracea*) and mustard (*Sinapis alba*) and it was found that AC and BC did not have a negative effect on plant growth [31]. In another study, the impact of aqueous biochar extracts produced from thermal conversion of sewage sludge on the growth of roots of *Lepidium sativum* was observed. Some of the biochar extracts had no significant effect on the growing root. However, one extract had a toxic effect that inhibited the root’s growth by 25% [10,32]. In another study, it was shown that toxic metals can be volatilized from contaminated soils by heating in non-oxidizing atmospheres like nitrogen, with removal occurring at different temperatures. Approximately 90% removal was achieved for mercury at 370 °C, cadmium at 550 °C, and zinc at 850 °C [33].

The toxic effects of aqueous extracts of biochars, derived from four different sources (mixed wood sievings, a mixture of paper sludge and wheat husks, and sewage sludge), on various plants (*Lepidium sativum*, *Lens culinaris Medikus*, *Cucumis sativus*, *Solanum lycopersicum*, *and Lactuca sativa*) were investigated. The effects of the biochar varied by plant species, where all biochars promoted the growth of L. *culinaris*, whereas wood biochar exhibited toxicity towards L. *sativum*, *C. sativus*, and *S. lycopersicum*. The highest toxicity, with a germination index (GI) of 49%, was observed in biochar made from paper sludge and wheat husks affecting L. *sativa* [10,30].

In addition to that, bacteria are frequently employed in toxicity tests because of their low cost, rapid growth, and sensitivity to a variety of toxic substances [28]. Gram-negative *Escherichia coli* (*E. coli*) is a widely used indicator for assessing environmental toxicity [27]. In some studies, the model organism *E. coli* was employed to evaluate the toxicity of activated carbon derived from pyrolyzed sugarcane-bagasse-containing silver nanoparticles, where the results indicated that these activated carbon composites possess the potential to impede the growth of the test organism, making them promising materials for applications in wastewater treatment and water purification. However, they exhibited adverse toxic effects on the aquatic organism *Hydra attenuata* and inhibited the root growth of *Lycopersicum esculentum* plants (tomatoes) as well [27].

Biochar derived from a mixture of sewage sludge with wheat straw, bark, or sawdust was prepared at pyrolysis temperatures of 300 or 600 °C. The toxicity of the samples was tested, and it was found to have no toxicity towards *Vibrio fischeri* [10,34]. Nevertheless, a negative effect of biochar on *Vibrio fischeri* was observed in some studies. Biochar produced from *Miscanthus* and wheat straw showed higher toxicity, whereas biochar made from wicker and coconut shell showed lower toxicity, inhibiting 40% and 12% of the *V. fischeri* luminescence [10,35]. *Vibrio fischeri* is a bioluminescent marine bacterium, which also serves as a model organism in toxicity tests due to its rapid and cost-effective cultivation and its high sensitivity to a wide range of toxic material [36]. When exposed to toxic substances, *V. fischeri’s* bioluminescence reduces, resulting in lower light intensity which can be used to determine the level of toxicity in the environment. However, its main drawback is its short potential exposure time (~15 min) which makes it less sensitive to substances with delayed toxic effects [28].

In this study, AC and BC samples were prepared from *Sargassum* sp. and characterized by employing various techniques. The potential adverse effects of the AC and BC samples were also assessed by examining their toxicity using *Sinapis alba* (white mustard) seeds and a bacteria-based toxicity test using *Escherichia coli* as a model organism. The AC and BC were compared to commercially available activated carbon samples. Furthermore, by using the AC and BC samples, polyurethane composites were prepared which can have various applications.

## 2. Materials and Methods

### 2.1. Preparation of the Activated Carbon (AC) and Biochar (BC)

Activated carbon and biochar are both porous carbonaceous materials derived from *Sargassum* sp., but they differ significantly in their production processes and pore structures.

The fresh *Sargassum* sp. (*Sargassum natans* and *Sargassum fluitans*) were collected in Guadeloupe at La Datcha beach [7]. Initially, the samples were sun-dried for 7 days to achieve dehydration, followed by placement in an oven set to 120 °C for at least 24 h. The dry samples were ground in a mixer grinder and then sieved from 0.4 to 1.5 mm, serving as the chosen material for producing activated carbon and biochar. The activated carbon was prepared by chemical activation and biochar by the pyrolysis of the *Sargassum* sp. [37].

#### 2.1.1. Activated Carbon Prepared by Chemical Activation

The *Sargassum* sp. was impregnated with 85% H_3_PO_4_ in a mass ratio of 3:1 (85% H_3_PO_4_/Sargassum) for 15 h (as the concentration of phosphoric acid increases, the porosity and surface area of the material significantly improved) [38]. After impregnation, the sample was heated in a tubular furnace at 5 °C/min until it reached a temperature of 600 °C. After two hours at the required temperature, the samples were naturally cooled. Throughout the process, a nitrogen flow of 80 mL/min was introduced into the furnace. Phosphoric acid (H_3_PO_4_, 85 wt%), was provided by Sigma-Aldrich (St. Quentin Fallavier, France).

#### 2.1.2. Biochar Sample Preparation

Biochar was produced through the pyrolysis of crushed *Sargassum* sp. in an advanced microwave oven (PYRO) at 1200 W (~650 °C) for 15 min under an inert nitrogen atmosphere. The resultant materials underwent washing initially with hydrochloric acid (5 M) followed by washing with deionized water until achieving a pH close to 7, and then they were dried.

In this study, four different samples were tested and compared (Table 1): commercial activated carbon (Sigma-Aldrich (St. Quentin Fallavier, France)), activated charcoal (Reference AC) (Norit^®^, Norit RBAA-3, rod, Sigma-Aldrich) and activated carbon prepared from *Sargassum* sp. by chemical activation with phosphoric acid, and biochar prepared from *Sargassum* sp. (elaborated using microwaves). The samples were identified as COMAC, AC, and BC, respectively.

### 2.2. Metal Content of the Samples

The metal content of the samples (COMAC, AC, BC, and Reference AC) was measured by using an inductively coupled optical emission spectrometer (ICP-OES, Varian 720 ES, Lab-EX, (Budapest, Hungary)) for 9 different metals (Ca, Cd, Co, Cu, Fr, Mg, Ni, Pb, and Zn), and to carry out the analysis microwave-assisted digestion using nitric acid (HNO_3_) was used.

### 2.3. Electrokinetic (Zeta) Potential Measurements

Zeta potential can be used to quantify the magnitude of the charge on the surface of a particle in a liquid. It is an important parameter in determining the stability of colloidal suspensions, as well as the interaction of particles with each other and with surfaces [39]. To compare the surface charge of studied activated carbon and biochar samples, zeta potential measurements were carried out on 0.05 g of powdered AC or BC suspended in 50 mL of distilled water.

### 2.4. Fourier Transform Infrared Spectroscopy (FTIR) Characterization and Specific Surface Area Analysis

The functional groups on the surface of the studied activated carbon and biochar were determined by using a (Bruker Optik GmbH Vertex 70 FTIR spectrometer, (Ettlingen, Germany)). All samples were investigated in potassium bromide pellets (0.05 g AC or BC in 0.16 g KBr).

The specific surface area was measured using nitrogen (N_2_) adsorption studies. A Sorptomatic 1990 series analyzer was employed to evaluate the N_2_ adsorption capacity at 77 K. Before the analysis, the samples were degassed at 250 °C for 17 h to remove any residual moisture. The specific surface area was calculated by applying a linear fit to the Brunauer–Emmett–Teller (BET) equation within the relative pressure range (P/P0) of 0.103 to 0.225 [37].

### 2.5. Toxicity Test of Activated Carbon and Biochar

To estimate the ecotoxicological effect of activated carbon and biochar on various organisms, two acute toxicity tests have been used. For the seed tests, commercially available *Sinapis alba* (white mustard) seeds were used, and the germination potential was verified by the Department of Environmental Protection, Nature Conservation, and Waste Management according to the Hungarian standard MSZ 6354-3 [40] before use. For the bacteria-based test, *Escherichia coli* (DH5a strain from the Department of Microbiology, Faculty of Science and Informatics, University of Szeged, Hungary) was employed as a model organism.

#### 2.5.1. Using *Sinapis alba* Seed (Mustard Seed) for an Acute Toxicity Test

Mustard seeds were exposed to the four different types of activated carbon and biochar: COMAC, AC, BC, and Reference AC. Three glass Petri plates were prepared for each sample by placing circular filter paper in the dishes which was then heat-sterilized along with the Petri dishes at 100 °C for 10 h before starting the experiment.

**Figure 1:**
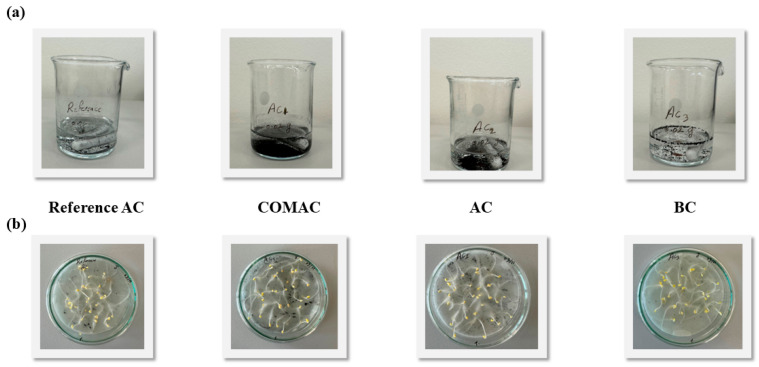
*Sinapis alba* seed test: (**a**) different types of AC/BC suspended in 20 mL of dilution water; (**b**) germinated seeds after incubation.

Then, 0.02 g of each type of AC or BC was suspended in 20 mL of special water-based solution (dilution water containing: 20 mM of CaCl_2_·2H_2_O, 5.0 mM of MgSO_4_, 7.5 mM of NaHCO_3_, and 0.75 mM of KCl) [41]. After that, 5 mL of the solution containing the studied samples was put into Petri dishes, and mustard seeds were distributed on the plates. Then, the plates were placed inside an incubator for 3 days in the dark at 20 °C. Thereafter, the root length of the seeds was measured to assess the effects of the different activated carbon and biochar samples on the organism (Figure 1).

#### 2.5.2. Liquid-Phase Toxicity Test Using the Bacterium *Escherichia coli* as a Model Organism

The bacterial tests involved the preparation of lysogeny broth (LB) liquid medium and LB plates solidified with agar (LB agar plates). LB liquid medium was first autoclaved, and 35 mL was put into Erlenmeyer flasks where it was used to make the starter bacterial suspension. To prepare LB agar plates the liquid LB medium supplemented with agar was poured into Petri plates and left to solidify overnight. The starter LB suspension was inoculated with *E. coli* and placed in an incubator shaker overnight at 37 °C and 160 rpm for bacterial growth. The concentration of bacteria was assessed using the OD_600_ measurement method. Based on the measured optical density of the overnight starter culture, a dilution was performed using a sterile LB medium to obtain a bacterial suspension with an optical density of OD_600_ = 0.15. The effectiveness of the initial dilution was reassessed to ensure accuracy. Subsequently, a ten-fold dilution series (ranging from 10^1^ to 10^6^) was prepared from the bacterial suspension with an OD_600_ = 0.15. The serial dilution was prepared to make the bacterial concentration measurable and to make it easier to count the colonies formed on the plate after incubation, where only plates containing colonies within the range of 30–300 CFU/mL should be considered [42]. This dilution was used to determine the initial bacterial concentration for the suspension used for further experiments.

The prepared bacterial suspension was used for two types of bacterial tests:

- Flask experiment

From 10^1^ to 10^6^ dilution series, the 10^4^ dilution was selected as the working bacterial solution and was used to inoculate the flasks containing the tested samples (0.02 g for each type of sample). First, 30 µL of the working solution was added to each flask (all activated carbon and BC samples were sterilized at 120 °C for 2 h before using them in bacterial tests), observing whether the liquid in the flask turns turbid after incubation (indicating normal bacterial growth) or remains clear (indicating toxicity).

The LB + *E. coli* flask (autoclaved LB medium, without any of the tested samples) was prepared to prove the presence of live cells in the utilized working bacterial solution and to show that the medium used is suitable for bacterial growth.

The flasks were incubated overnight at 37 °C in a shaker incubator (160 rpm). After the incubation of the prepared flasks, the overnight cultures were diluted with a sterile LB medium by applying a 10^1^–10^6^ dilution for all flasks. As mentioned before, a serial dilution was used to optimize the bacterial concentration to the level needed for the particular test which makes it easier to count the bacteria. Then, 100 µL from the 10^6^ dilution of every flask (LB + *E. coli,* LB + Reference AC + *E. coli,* LB + COMAC + *E. coli,* LB + AC + *E. coli,* LB + BC + *E. coli*) was spread on the top of the LB agar (3 plates for each tested sample) and after another 12–16 h incubation of the plates, the colony count was determined.

- Plate test

AC and BC samples were also tested by spreading the bacterial working suspension on the top of the agar plates. When testing bacterial liquid suspensions on agar plates, it is important to select a volume that evenly and completely covers the surface of the plate, allowing the liquid to dry relatively quickly. If drying takes too long, it increases the risk of contamination, while using too little suspension may cause it to dry before it can be evenly spread. Uneven distribution can result in bacterial colonies forming too closely, making it difficult or impossible to accurately count cells and determine cell concentration. Based on previous in-house experience, 100 µL of bacterial suspension was found to meet these criteria and employed in this case as well. After evenly spreading bacterial suspension on the plates, 0.02 g of different types of samples (COMAC, AC, BC, and Reference AC) was placed on the top of the plates. Following that, all the plates were placed in an incubator overnight at 37 °C, and the potential formation of a zone of inhibition was tested.

### 2.6. Preparation of Polymer Composites, Polyurethane Foam/Activated Carbon, and Biochar (PUF/AC and PUF/BC)

The production of flexible polyurethane foam involves several precursors and additives. Specifically, in the current study, Ongronat TR4040 (a mixture of methylene-diphenyl-diisocyanate isomers, Wanhua-BorsodChem, Kazincbarcika, Hungary) and Ongropur FFP-303 (a polyether-type polyol premix, Wanhua-BorsodChem) were used as the main precursors. Catalysts, blowing agents, and surfactants (DABCO 33LV, Jeffcat ZF-22, Tegoamin DEOA 85, and Tagostab B4113) are also mixed into the base polyol system (Alcupol F2831 (100 pphp) from Repsol in Madrid, Spain) (Table 2).

The polymer composite samples were prepared with 1.0 isocyanate indices (NCO-1.0). The isocyanate index represents the ratio of the total NCO groups to the total number of the OH groups in the polyol premix, including any additives (such as water) present in the reaction mixture.

The composite materials (PUF/AC-PUF/BC) were prepared by using the Foam Qualification System FOAMAT^®^ 285 by adding AC or BC to the polyol with three different percentages (1%, 2%, and 3%) from each type of activated carbon and biochar (COMAC, AC, BC, and Reference AC). A control foam was also prepared (PUF) without adding AC or BC and used in comparison with the other samples. These percentages were chosen based on previous studies to examine how varying amounts of activated carbon and biochar affect the mechanical strength and flexibility of the foams.

### 2.7. Mechanical Measurements

Mechanical tests on the polymer composite samples were carried out according to ASTM D3574 (Test Method C) [43]. The compression force deflection (CFD) test is used to determine the force required to achieve 50% compression over the entire surface of a sample [44]. All samples were cut into a circular shape with 35 mm thickness which is in accordance with ASTM D3574 as the thickness is between 25 mm and 50 mm. The samples were compressed to 50% of their initial height (Figure S1). The experiment was carried out three times to compare with the other samples, and the average and standard deviation for all measurements were calculated.

### 2.8. Acoustic Measurements

To measure the acoustic performance of the composite samples in the higher frequency range, the impedance tube technique was used to determine the sound absorption coefficient (α). In this study, the AED 1000 AcousticTube^®^ impedance tube (Gesellschaft für Akustikforschung Dresden GmbH, Dresden, Germany) with three microphones was used which is usually utilized to determine the sound absorption coefficient, based on the transmission function method according to EN ISO 10534–2 [45] and ASTM E1050 [46] (Figure S2). The internal diameter of the tube is 30 mm, and the corresponding frequency is 250–5000 Hz. The composite samples with a thickness of 35 mm were placed at the end of the tube and the incident sound wave was vertical to the surface of the sample.

## 3. Results and Discussion

### 3.1. Metal Content Measurement of Carbonaceous Materials

The metal content of activated carbon and biochar was tested to identify the levels of heavy metals in these samples, which could pose environmental or health risks and contribute to the potential toxicity of these materials and any composites made from them.

The concentrations of various metals in the activated carbon/biochar samples were analyzed using ICP-OES measurements, and the results were compared in terms of *m*/*m*%. The predominant metal observed in the AC/BC samples was calcium (Ca), with concentrations of 0.135, 0.951, and 0.635 *m*/*m*% for COMAC, AC, and Reference AC, respectively. Calcium was also present in BC, but at a lower concentration compared to magnesium (Mg), which showed the highest concentration in the biochar sample with a value of 1.246 *m*/*m*%. The heavy metals lead (Pb) and cadmium (Cd) exhibited the lowest concentration, with values < 0.001% for all samples (Table 3). Additionally, other metals such as Co, Cu, Fe, Ni, and Zn were also present in all samples but only at low concentrations. The higher presence of calcium in activated carbon produced via phosphoric acid chemical activation and the presence of magnesium in biochar by pyrolysis primarily arise from the inherent calcium and magnesium content in the precursor material. During the activation process, calcium interacts with phosphoric acid to form stable, non-volatile compounds retained within the activated carbon structure. Biochar was produced through the pyrolysis of crushed *Sargassum* sp. at 600 °C which begins to volatilize at temperatures above 800 °C, explaining its higher concentration in the biochar [47].

### 3.2. Electrokinetic (Zeta) Potential Measurements of AC/BC Samples

**Figure 2:**
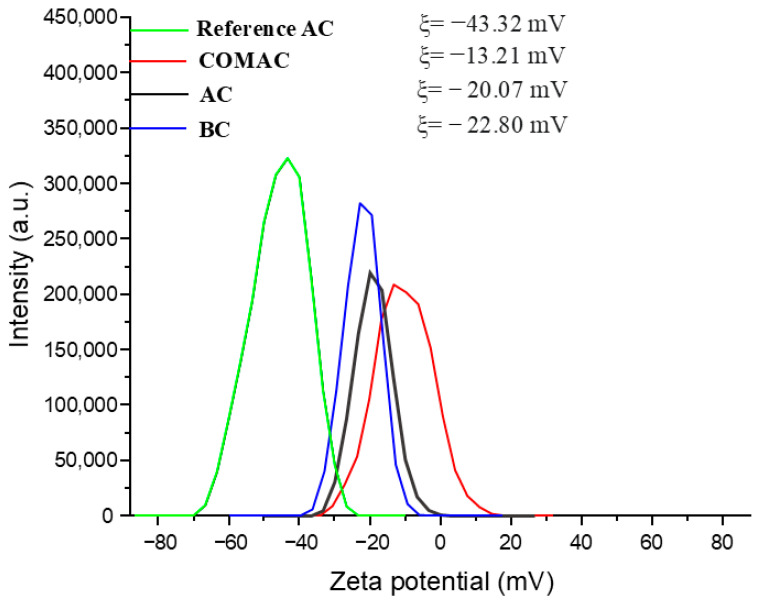
Zeta potential distribution of the four different activated carbon/biochar samples.

The electrokinetic properties of the AC samples differ due to the various activation processes, which create oxygenated functional groups on the surface [48]. The negative charge of the AC/BC particles can be attributed to the deprotonation of functional groups (e.g., carboxyl) [49,50]. The lowest zeta potential value was found in the case of Reference AC, with a value of −43.32 mV which indicates that the dispersion of the sample has good stability (Figure 2, Table S1).

The second lowest zeta potential was associated with BC, with a value of −22.80 mV. This indicates that the BC particles have a strong negative surface charge, which will help to repel each other, keeping the particles well-dispersed and preventing aggregation by causing electrostatic repulsion between the particles. This property is crucial for maintaining uniform dispersion, which enhances the effectiveness of AC or BC in applications such as water purification where uniform distribution is needed for optimal performance or when incorporating these samples into composite materials where preventing aggregation improves interaction within the matrix and enhances the overall properties of the final product.

### 3.3. Infrared Spectroscopy Characterization of the Studied Samples

**Figure 3:**
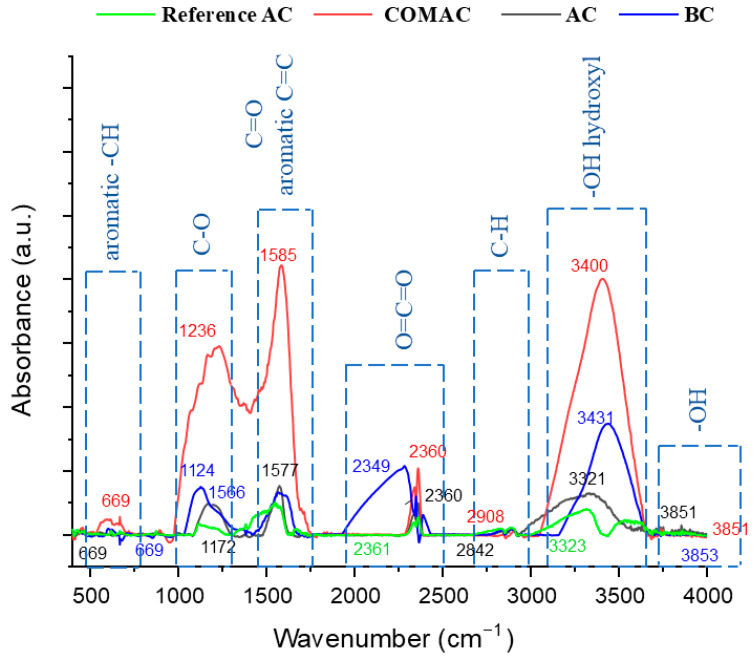
FTIR spectra of the four different activated carbon/biochar (COMAC, AC, BC, and Reference AC) samples.

To identify the functional groups in the AC/BC samples, FTIR was used [51]. All studied samples have the same tendency, but the highest intensities were determined in the case of COMAC. The spectra of all samples contained peaks at 650 cm^−1^ attributed to aromatic C–H bending [52]. The peaks within the range of 1126–1236 cm^−1^ correspond to C-O stretching in alcohols and phenols [51,52]. The band observed at about 1566 cm^−1^ may be associated with aromatic and C=O (carbonyl) stretching vibration [52]. The CO_2_ infrared standard peak (2349 cm^−1^) splits into two peaks (2330 cm^−1^ and 2360 cm^−1^) caused by the asymmetric and symmetric stretching vibrations of CO_2_ (peak at 2349 cm^−1^ is attributed to O=C=O) [53]. Very small peaks are found from 2842–2908 cm^−1^, which are associated with the symmetric and asymmetric stretching vibration of aliphatic and aromatic C-H [50,51]. The functional group represented by the bands about 3400 cm^−1^ is related to the stretching vibration of the surface hydroxyl group -OH [54]. The stretching vibration band of hydroxyl group –OH could be observed at 3851 cm^−1^ [50] (Figure 3).

Surface area measurement is applied to activated carbon and biochar, where a larger surface area indicates a more porous structure, enhancing the materials’ effectiveness in applications such as adsorbing the contaminants from water and soil.

The specific surface area of the samples was measured using the Brunauer–Emmett–Teller (BET) method with nitrogen adsorption at 77 K (Table 4). By comparing all the samples, AC samples showed the largest surface area of 1695 m^2^/g, indicating a highly porous structure. In contrast, biochar showed the lowest value of 854 m^2^/g. The Reference AC had a slight reduction in BET surface area, being >1500 m^2^/g, but still had high porosity (according to the manufacturer data). The COMAC sample had a moderate specific surface area of 1120 m^2^/g.

### 3.4. Toxicity Test

#### 3.4.1. Acute Toxicity

##### Seed Test for Activated Carbon and Biochar

Evaluating seed germination and measuring root length are simple and widely used ecotoxicological methods for assessing the toxicity of activated carbon and biochar [30]. *Sinapis alba* is considered an effective organism for detecting potential toxic effects of substances like activated carbon and biochar [31,55] and the test is considered valid if 90% of the seeds germinate on the plate that only contains the dilution water, and if the root length of seeds is similar to those observed in the reference sample (in this case Reference AC) the tested samples are considered non-toxic [56].

The root lengths and percentages for all samples have been measured (Table 5 and Table S2) and compared to Reference AC. The root length of *Sinapis alba* seeds for the COMAC and AC plates was within a similar range to those of the Reference AC plates. The root length was 4.8 and 4.9 cm (Table 5) with percentages of 104.3%, and 106.5%, respectively, compared to Reference AC.

It can be seen from the results that the length of the root slightly decreased in the case of biochar in comparison to Reference AC, and the root length was around 91.3% which is a ~8.7% reduction (Table S2). The toxicity of biochar to plants can vary significantly depending on the feedstock used for producing it, as well as the pyrolysis temperature [10].

**Figure 4:**
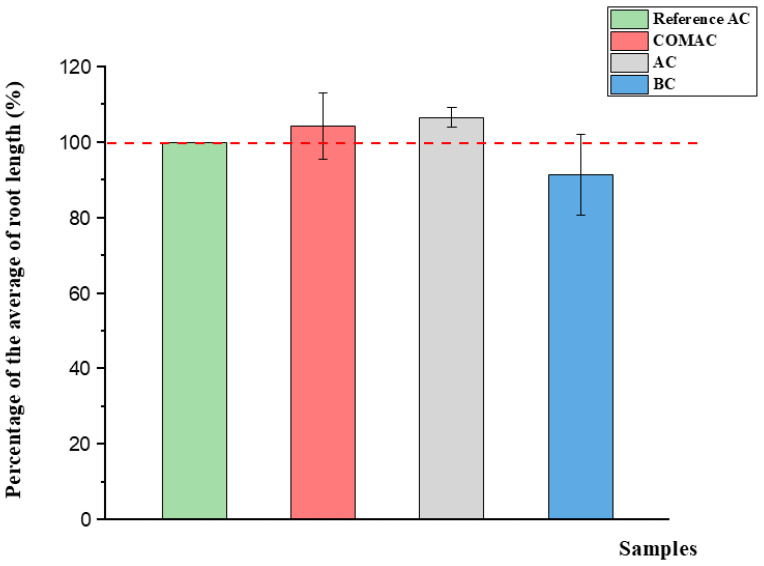
Percentage of the length of the growing root *of Sinapis alba* (white mustard) seeds exposed to COMAC (commercial activated carbon), AC (activated carbon was prepared from *Sargassum* sp. by chemical activation), and BC (biochar prepared from *Sargassum* sp. by pyrolysis) compared to the growing roots exposed to the Reference AC (Norit^®^ activated charcoal). The red dotted line refers to the level of germination seeds for the samples in comparison to the Reference AC. Bacterial Test for Activated Carbon and Biochar.

Comparing all tested samples, the highest germination rate for mustard seeds was observed in the case when AC was added which increases the average by 106.5% which is higher than for COMAC and BC (Figure 4, Table 5 and Table S2).

Overall, all samples contributed to growing the mustard seeds, but the average length of the roots varies among them. This could be attributed to factors such as particle size in the samples and material composition. Activated carbon and biochar can enhance growth and seed germination because of their metal content and the partial bioavailability of carbon within their porous structure [10]. Generally, the metal content of activated carbon from commercial COMAC, AC, and biochar produced from biomass does not have a negative effect on the mustard seeds’ growth, conversely, it has a positive effect which helped in growing the seeds, especially when compared with plates containing only dilution water where the root length was 3.6 ± 0.3 cm.

Toxicity tests using *E. coli* were also performed to make sure the samples were safe and suitable for a wide range of applications such as adsorbing pollutants in water [26], soil amendment, and as filler in composite materials [24].

**Figure 5:**
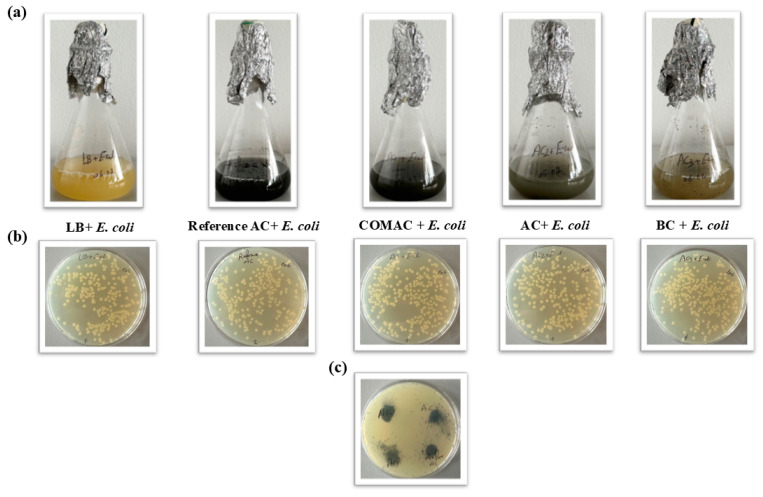
Bacteria-based toxicity tests in liquid phase: (**a**) LB flasks containing AC/BC samples with *Escherichia coli* liquid medium, (**b**) plates with *Escherichia coli* grown in LB solid medium, for colony counting, and (**c**) plate with spread of 100 µL from bacterial suspension with AC/BC samples.

Colonies can only form if no toxic substances are released from the tested samples, confirming that the sterilization process (at 120 °C for 2 h) was efficient in preventing the release of any toxins that could impede the growth of the bacteria (Figure 5).

When all AC- or BC-containing flasks were sterilized and then inoculated with *E. coli*, the growth of bacteria was detected by counting the number of colonies on the plates. It was found that in the case of both the Reference AC + *E. coli* plate and plates made from the flasks with various AC or BC samples coincubated with *E. coli*, the colony counts fell within a similar range, specifically between 2.7 × 10^9^ and 3.1 × 10^9^ CFU/mL, showing a similarity in the growth and viability of bacteria. This observation assumes that only non-toxic materials were released from the various AC or BC samples into the bacterial growth medium (Table 6).

The possible toxicity of tested samples was measured in another technique as explained previously by spreading 100 µL from a bacterial suspension containing *E. coli* over the agar plate and adding a certain amount of different tested samples. In this method, a clear zone (the zone of inhibition) around the material placed on the plate could appear or not. A clear zone indicates toxicity, meaning the tested material has inhibited or killed the bacteria in that area. If the material is not toxic, the bacteria will grow normally around the tested samples, and no clear zone will be visible. As no clear zone was noticed around the samples, the tested materials are not toxic (Figure 5c).

According to the toxicity test using *E. coli*, no reduction in bacterial viability by activated carbon and biochar was observed. As a result, it was determined that all the samples were non-toxic.

This result is in agreement with other studies where activated carbon was prepared from coffee waste residue and no ecotoxicity towards the bacterium *Escherichia coli* was detected [57]. Since the tested materials, activated carbon (AC) and biochar (BC), are non-toxic, they can be applied in various applications, including composite preparation, water purification, removal of heavy metals from water, and soil amendment.

### 3.5. Mechanical Tests of the Prepared Polymer Composites

As the studied samples were not toxic, and thus can be employed in wider applications, polyurethane composites were prepared by using the AC and BC samples, and their properties have been tested and compared. Mechanical tests are commonly used in the evaluation of several physical properties, including density, compressive strength, tensile strength, etc. Mechanical properties are usually examined by compression testing, particularly for polymer foams [16]. A compression test was carried out on four composite samples, PUF/Reference AC, PUF/COMAC, PUF/AC, and PUF/BC, and compared to control PUF. The samples were pressed at 50% of their height and held there for 60 s, and it was repeated three times to calculate the average force.

**Figure 6:**
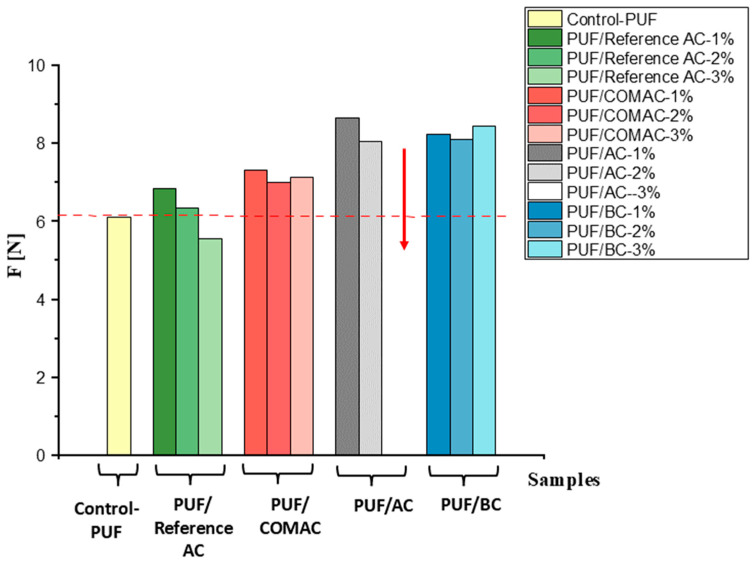
Average compression force (F) deflection of composite samples PUF/Reference AC, PUF/COMAC, PUF/AC, PUF/BC, and the control PUF sample. The red arrow refers to the PUF/AC at 3% which was not possible to measure. The red dotted line refers to the level of the compression force of all composite samples compared to Control-PUF.

The mechanical properties of the polyurethane foam composites reinforced with 1, 2, or 3% AC or BC were determined (Figure 6, Table S3). It is noticed that by increasing the amount of AC/BC in the sample, the hardness of the composite increased, such as in the case of PUF/BC where the average compression force also slightly gradually increased from 8.24 N to 8.45 N (Figure 6) which is in agreement with other findings where adding 0.1 wt% BC from eggshell waste to PUF increased the compressive strength by 4.02% compared to unfilled PUF. This can be explained by enhanced hydrogen bonding and dispersion between BC and the polymer matrix [58].

In the case of PUF/AC, at a higher percentage (3%) the composite’s structure becomes fragile and adding this amount of AC caused destruction in the cellular structure [16] and achieved a high density of 60.3 g/L [59]. It can be concluded that a maximum of ~2% of AC can be added to PUF, because after that shrinkage in the sample will occur and the sample will lose its flexibility (labeled with red arrow in Figure 6). Similar results were presented previously where, when the amount of powder-activated carbon in the polyurethane foam exceeded 5.81% by weight, the composite sample became fragile [16].

Overall, adding activated carbon and biochar may contribute to an increase in compressive strength of composite samples, resulting in higher forces required to compress them. This effect can be attributed to the reinforcing properties of activated carbon/biochar, which may improve the mechanical performance of the composite material in compression due to the interaction between the AC/BC and the polyurethane matrix, as demonstrated by comparison with the control PUF which has the lowest compressive force at 6.12 N. These results are in good agreement with other findings where the mechanical properties of rubber were enhanced by adding leaf biomass/AC due to the network formation resulting from the interaction between the filler and rubber, where it was found that the tensile strength was increased by approximately 8% and there was a 40% increase in Young’s modulus [60].

All in all, the composite material prepared from activated carbon and biochar derived from a natural source (*Sargassum* sp.) can improve the mechanical properties of polyurethane foam. Both materials have porous structures, with a large specific surface area. AC typically exhibits a larger surface area of 1695 (m^2^/g) due to more extensive activation processes. This large surface area can enhance the interaction between the filler and PU matrix, potentially improving the compressive strength of PUF composite. Biochar, with its structure, may contribute to improved compression strength and dimensional stability in PU foam. Furthermore, the functional groups on the AC and BC surfaces can form strong interfacial bonds with the PU matrix, leading to better mechanical performance. This improvement is probably a result of the enhanced compatibility between AC/BC and the PU matrix, which facilitates the creation of urethane bonds between the isocyanate and the AC/BC fillers.

Specifically, the compressive force increased for PUF/BC at 3% BC content by approximately 2.88 N compared to PUF/Reference AC and by 1.32 N compared to PUF/COMAC (Figure 6, Table S3).

In contrast, in PUF/Reference AC, when increasing the amount of activated carbon by 3%, the compressive strength decreased. This reduction is attributed to the agglomeration of activated carbon particles at higher concentrations, leading to the weakening of the structure and, thus, it is unable to withstand the applied load [58].

### 3.6. Acoustic Test of the Prepared Polyurethane Composites

**Figure 7:**
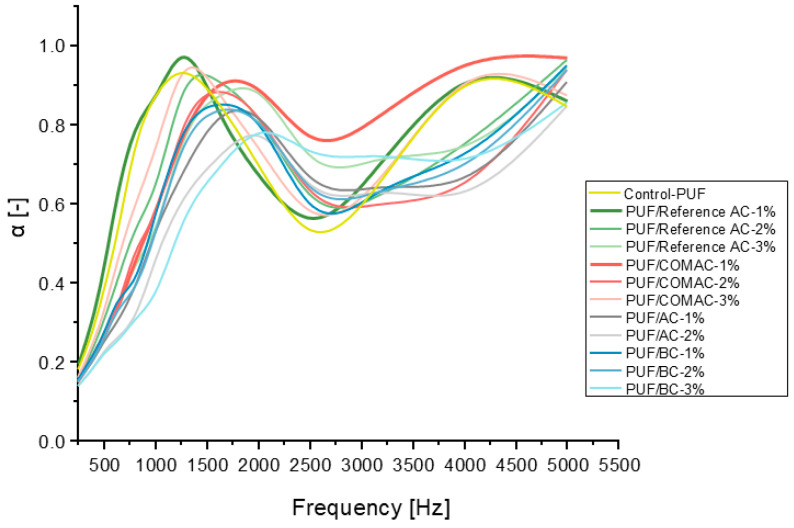
Sound absorption coefficient (α) for composite samples PUF/Reference AC, PUF/COMAC, PUF/AC, PUF/BC and the control sample PUF.

The sound absorption coefficients of the prepared composite samples containing four different types of activated carbon and biochar samples (COMAC, AC, BC, and Reference AC) and the control PUF with NCO-index-1.0 were measured by using an impedance tube. Sound absorption coefficients vary between 0 and 1, and the higher the coefficient, the more effective the material is at absorbing sound. When sound waves strike a porous material, the air moves across its surface and through its pores and it starts to vibrate. This causes a loss of some of the air’s original energy, as it is converted to heat due to thermal and viscous losses along the internal pore walls and the material’s tortuous structure [23]. As can be seen, the maximum sound absorption of the studied samples occurs at certain frequencies, and all curves show a peak within the measured frequency range (Figure 7). The control PUF without added AC or BC absorbs the incident acoustic waves with a 0.93 absorption coefficient value at a peak mid-frequency of 1250 Hz.

However, the sound absorption coefficients for control PUF are 0.69 and 0.84 at frequencies of 2000 Hz and 5000 Hz, which are lower than other samples. For example, after including AC or BC in the PUF matrix, the sound absorption properties are enhanced at the same frequency, where the enhancement is attributed to its large surface area and the porosity of composites (Figure 7, Table S4) [23].

Also, it can be seen that, by increasing the frequency from 2000 to 5000 Hz, the sound absorption performance increased for all samples. Notably, the 1% PUF/COMAC composite sample exhibited the highest sound absorption coefficient in the range of 2000–5000 Hz compared to all other composite samples. The PUF/BC composite sample at 1% and 2% showed better sound absorption than PUF/AC at the same frequency range, achieving a maximum sound absorption coefficient of 0.95 at 5000 Hz for BC content of 1% (Table S4). Adding biochar into the polyurethane foam (PUF) matrix significantly enhances the sound absorption properties, primarily due to the porous structure of biochar. This porous nature allows sound waves to be dissipated, leading to effective sound attenuation. Additionally, biochar possesses a negative surface charge that helps to prevent the aggregation of biochar particles within the composite. As a result, the biochar particles remain well-dispersed throughout the PUF matrix, creating a homogeneous structure. This uniform distribution facilitates the effective absorption of sound waves within the composite material, thereby improving its overall acoustic performance.

It can be concluded that adding AC or BC to the polyurethane matrix can improve the acoustic properties in the range of 2000–5000 Hz. It should be noted that this composite foam effectively absorbs sound waves in the frequency range of 2000–5000 Hz, to which human hearing is particularly sensitive [25].

## 4. Conclusions

Activated carbon and biochar are versatile substances that find wide use in various applications. In this work, four different samples of activated carbon and biochar (COMAC, Reference AC, AC, and BC) have been studied. FTIR spectra revealed that all the samples have the same functional groups, carbonyl and hydroxyl groups were identified on the surface of the activated carbon and biochar samples, and the activated carbon from *Sargassum* sp. showed the largest surface area with 1695 m^2^/g. The zeta potential of the Reference AC and biochar was the lowest, −43.23 mV and −22.80 mV, which indicates that the dispersion of these has better stability than that of other samples, with −13.21 mV and −20.07 mV for COMAC and AC, respectively. The primary metal detected in the AC/BC samples was Ca for COMAC, AC, and Reference AC. Although calcium was present in the BC sample, it was found in lower concentrations compared to magnesium. Toxicity tests were also carried out to determine the environmental impact of the prepared activated carbon and biochar samples. By using *Sinapis alba* seeds as model organisms it was found that COMAC and AC samples have the same effect on the average root length compared to Reference AC, while a reduction in root length was observed in the case of BC with a decrease of ~8.7%. In addition to that, the toxicity of the samples was also determined by using *Escherichia coli* as a model organism, where there was no observed decrease in bacterial viability in the presence of the studied samples. Thus, it was concluded that all investigated carbonaceous materials are non-toxic and that it is safe to employ them in various applications. Therefore, AC and BC were incorporated into polyurethane foam with different percentages (1%, 2%, and 3%), polymer composites were prepared, and the mechanical and acoustic properties of the composite foams were measured. During the mechanical tests of PUF AC/BC composites, it was noticed that adding the AC or BC can increase the compression strength for all the samples compared with control polyurethane foam. However, in the case of PUF/AC samples, the maximum recommended amount of AC is ~2% because shrinkage was observed in the composite’s structure when 3% was added and it became brittle. The acoustic measurements revealed similar effects, where adding AC or BC enhanced the sound adsorption coefficient (α) for all composite materials when compared to the control PUF in the frequency range of 2000–5000 Hz. All in all, it can be concluded that by using *Sargassum* sp. as raw materials for AC or BC preparation, valuable products can be achieved which are applicable to enhance the properties of polymer composites.

## Figures and Tables

**Table 1:** Source of the carbonaceous materials.

Sample	Source
Reference AC	Norit RBAA-3, rod, Sigma-Aldrich
COMAC	Commercial activated carbon
AC	Prepared from *Sargassum* sp. chemical activation
BC	Prepared from *Sargassum* sp. by pyrolysis process

**Table 2:** Formulation of the polyurethane foam and percentage of AC/BC in PUF matrix.

		NCO-Index-1.0	
Percentage of AC or BC	Weight of AC or BC	Polyol Premix	Isocyanate
		Ongropur FFP-303 [g]	Ongronat TR4040 [g]
1%	0.6	36.34	23.06
2%	1.2	35.75	23.06
3%	1.8	35.14	23.06

**Table 3:** Comparison of the metal content of activated carbon/biochar samples (COMAC, AC, BC, and Reference AC) determined by using ICP-OES.

Sample	Concentration of Metals in *m*/*m*%								
	Ca	Cd	Co	Cu	Fe	Mg	Ni	Pb	Zn
Reference AC	0.635	<0.001	<0.001	0.001	0.020	0.1824	<0.001	<0.001	0.0036
COMAC	0.135	<0.001	<0.001	<0.001	0.047	0.068	0.0011	<0.001	0.0064
AC	0.951	<0.001	<0.001	0.0016	0.048	0.097	0.0015	<0.001	0.0146
BC	0.969	<0.001	<0.001	<0.001	0.019	1.246	0.002	<0.001	0.0043

**Table 4:** Specific surface area of the samples.

Sample	BET Surface Area (m^2^/g)
Reference AC	>1500
COMAC	1120
AC	1695
BC	854

**Table 5:** The average root lengths of the growing seeds without (e.g., Dilution water-1) and in contact with the studied samples (DARC AC, AC, BC, and Reference AC).

Sample	Root Length (cm)
Dilution water-1	3.9
Dilution water-2	3.6
Dilution water-3	3.3
Average Dilution water	3.6 ± 0.3
Reference AC-1	4.8
Reference AC-2	4.7
Reference AC-3	4.5
Average Reference AC	4.6 ± 0.2
COMAC-1	5.0
COMAC-2	4.4
COMAC-3	5.0
Average COMAC	4.8 ± 0.3
AC-1	5.0
AC-2	4.9
AC-3	4.9
Average AC	4.9 ± 0.1
BC-1	4.2
BC-2	4.7
BC-3	3.7
Average BC	4.2 ± 0.5

**Table 6:** Colony forming units (CFU) for all samples (COMAC + *E. coli*, AC + *E. coli*, BC + *E. coli*, Reference AC + *E. coli*).

Sample	CFU/mL
LB + *E. coli*	2.7 × 10^9^
Reference AC + *E. coli*	3.0 × 10^9^
COMAC + *E. coli*	3.1 × 10^9^
AC + *E. coli*	3.2 × 10^9^
BC + *E. coli*	2.9 × 10^9^

## Data Availability

The original contributions presented in the study are included in the article/Supplementary Materials, further inquiries can be directed to the corresponding authors.

## Supplementary Materials

The following supporting information can be downloaded at: https://www.mdpi.com/article/10.3390/polym16202914/s1, Figure S1: Schematic illustration of compression test. Figure S2: AED 1000 AcousticTube^®^ impedance tube with three microphones. Table S1: Electrokinetic (zeta) potential of the activated carbon/biochar samples (COMAC, AC, BC, and Reference AC). Table S2: Percentage of the length of the growing root exposed to COMAC, AC, and BC compared to those exposed to the Reference AC sample. Table S3: Compression force deflection test results for polyurethane composite samples and control PUF. Table S4: Sound absorption coefficients (α) of studied composite samples PUF/Reference AC, PUF/COMAC, PUF/AC, PUF/BC and control PUF at different ranges of frequency.
