# Supplementary material for: Activated Carbon and Biochar Derived from Sargassum sp. Applied in Polyurethane-Based Materials Development
Source: Polymers (Basel). 2024 Oct 16;16(20):2914. doi: 10.3390/polym16202914 (PMC11510917; doi:10.3390/polym16202914)
Supplement: Supplementary file 1 [file polymers-16-02914-s001.zip › polymers-3263230-supplementary.pdf]

polymers

Activated Carbon and Biochar Derived from *Sargassum* sp. Applied in Polyurethane…

Julie Mallouhi [1,2], Miklós Varga[1,2], Emőke Sikora [1,2], Kitty Gráczer [1], Oliver Bánhidi [1], Sarra Gaspard[3], Francesca Goudou[3] , Béla Viskolcz [1,2] ,Emma Szőri-Dorogházi [2,*] Béla Fiser[1,2,4,5,*]

[1] Institute of Chemistry, University of Miskolc, 3515 Miskolc-Egyetemváros, Hungary
[2] Higher Education and Industrial Cooperation Centre, University of Miskolc, 3515 Miskolc-Egyetemváros, Hungary;
julie.mallouhi@gmail.com (J.M.); miklos.varga@uni-miskolc.hu (M.V.); emoke.sikora@uni-miskolc.hu (E.S.); graczkit22@gmail.com (K.G.) ; banhidi9@t-online.hu (O.B.) ; sarra.gaspard@univ-antilles.fr (S.G.) ; francesca.goudou@univ-antilles.fr (F.G.); bela.viskolcz@uni-miskolc.hu (B.V.); emma.szdoroghazi@uni-miskolc.hu (E.S.) ;bela.fiser@uni-miskolc.hu (B.F.).
[3] Laboratory COVACHIM-M2E, EA 3592 Université des Antilles, BP 250, 97157 Pointe à Pitre Cedex, France.
[4] Department of Biology and Chemistry, Ferenc Rakoczi II Transcarpathian Hungarian College of Higher Education, 90200 Beregszász, Ukraine
Transcarpathia, Ukraine
[5] Department of Physical Chemistry, Faculty of Chemistry, University of Lodz, 90-236 Lodz, Poland

* Correspondence: bela.fiser@uni-miskolc.hu and emma.szori-doroghazi@uni-miskolc.hu

Supporting Information

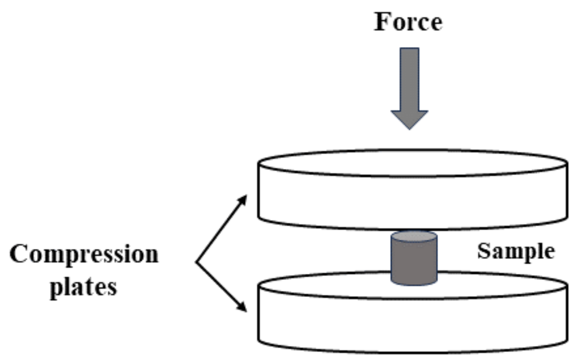

Figure S1. Schematic illustration of compression test

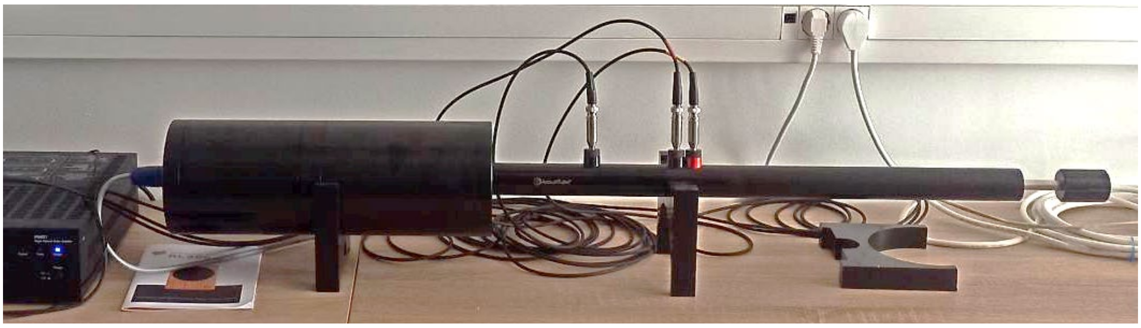

Figure S2. AED 1000 AcousticTube® impedance tube with three microphones

Table S1. Electro-kinetic (zeta) potential of the activated carbon/biochar samples (COMAC, AC, BC, and Reference AC)

Sample	Zeta potential (mV)
Reference AC	-43.32
COMAC	-13.21
AC	-20.07
BC	-22.80

Table S2. Percentage of the length of the growing root exposed to COMAC, AC, and BC compared to those exposed to the Reference AC sample

Sample	Percentage (%)
Reference AC	100.0
COMAC	104.3±7.5
AC	106.5±1.7
BC	91.3±11.1

Table S3. Compression force deflection test results for polyurethane composite samples and control PUF

NCO-Index-1.0	Percentage of AC/BC%	number of measurements	$F_{50\%}$ (N)	$F_{50\%}$ (N), Average	h_0 (mm)	Density g/l
PUF/Reference AC	1%	PUF/Reference AC-1	7.101	6.85	38.291	49.5
		PUF/Reference AC-2	6.740		37.982	
		PUF/Reference AC-3	6.711		38.019	
	2%	PUF/Reference AC-1	6.567	6.36	39.043	49.1
		PUF/Reference AC-2	6.351		38.783	
		PUF/Reference AC-3	6.175		38.597	
	3%	PUF/Reference AC-1	5.726	5.57	40.835	49.8
		PUF/Reference AC-2	5.465		40.663	
		PUF/Reference AC-3	5.528		40.335	
PUF/COMAC	1%	PUF/COMAC-1	7.497	7.32	38.825	49.1
		PUF/COMAC-2	7.306		39.269	
		PUF/COMAC-3	7.183		39.269	
	2%	PUF/COMAC-1	6.626	6.99	41.041	48.9
		PUF/COMAC-2	7.200		39.220	
		PUF/COM AC-3	7.153		37.482	
	3%	PUF/COMAC-1	7.534	7.13	37.585	49.3
		PUF/COMAC-2	6.933		39.059	
		PUF/COMAC-3	6.939		37.249	
PUF/AC	1%	PUF/AC-1	8.648	8.66	39.760	49.6
		PUF/AC-2	8.666		38.903	
		PUF/AC-3	8.679		38.437	
	2%	PUF/AC-1	7.973	8.05	39.270	49.3
		PUF/AC-2	8.102		38.188	
		PUF/AC-3	8.093		37.733	
	3%	PUF/AC-1	-		-	60.3
		PUF/AC-2				
		PUF/AC-3				
PUF/BC	1%	PUF/BC-1	8.258	8.24	40.418	50.7
		PUF/BC-2	8.208		40.018	
		PUF/BC-3	8.278		39.415	
	2%	PUF/BC-1	7.966	8.10	39.976	50.8
		PUF/BC-2	8.179		39.360	
		PUF/BC-3	8.164		38.551	
	3%	PUF/BC-1	8.806	8.45	38.436	50.2
		PUF/BC-2	8.648		37.915	
		PUF/BC-3	7.923		38.097	
Control PUF		PUF-1	6.051		36.478	50.3

	PUF-2	6.187	6.12	35.479
	PUF-3	6.149		35.172

Table S4. Sound absorption coefficients (α) of studied composite samples PUF/Reference AC, PUF/COMAC, PUF/AC, PUF/BC, and control PUF at different ranges of frequency.

**Sample with % of AC/BC in PUF**	**1/3 octave frequency (Hz)**										
	**500**	**630**	**800**	**1000**	**1250**	**1600**	**2000**	**2500**	**3150**	**4000**	**5000**
	**Sound absorption coefficient (α)**										
Control PUF	0.38	0.52	0.73	0.87	0.93	0.86	0.69	0.53	0.64	0.89	0.84
PUF/Reference AC 1%	0.44	0.62	0.78	0.87	0.97	0.84	0.67	0.56	0.69	0.90	0.86
PUF/Reference AC 2%	0.30	0.40	0.52	0.64	0.86	0.91	0.81	0.62	0.61	0.74	0.96
PUF/Reference AC 3%	0.27	0.33	0.43	0.53	0.74	0.86	0.87	0.72	0.71	0.74	0.94
PUF/COMAC 1%	0.27	0.33	0.45	0.58	0.76	0.89	0.88	0.77	0.81	0.95	0.97
PUF/COMAC 2%	0.26	0.34	0.47	0.57	0.77	0.88	0.81	0.63	0.59	0.65	0.94
PUF/COMAC 3%	0.32	0.45	0.59	0.73	0.92	0.88	0.74	0.58	0.65	0.90	0.87
PUF/AC 1%	0.24	0.30	0.38	0.52	0.66	0.81	0.81	0.66	0.64	0.66	0.91
PUF/AC 2%	0.22	0.25	0.31	0.45	0.59	0.71	0.77	0.64	0.62	0.63	0.85
PUF/AC 3%	-	-	-	-	-	-	-	-	-	-	-
PUF/BC 1%	0.27	0.35	0.41	0.57	0.76	0.84	0.80	0.60	0.62	0.72	0.95
PUF/BC 2%	0.25	0.32	0.38	0.52	0.72	0.83	0.80	0.64	0.62	0.70	0.94
PUF/BC 3%	0.21	0.25	0.30	0.37	0.54	0.68	0.77	0.73	0.72	0.71	0.86
